# Supplementary figures and images for: Cold induces brain region-selective cell activity-dependent lipid metabolism
Source: eLife. 2025 Jan 30;13:RP98353. doi: 10.7554/eLife.98353 (PMC11781799; doi:10.7554/eLife.98353)

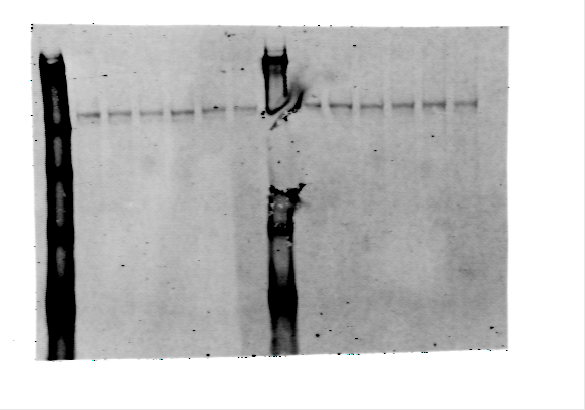

Supplement: Figure 4—source data 1. [file elife-98353-fig4-data1.zip › Figure 4A (HSL)-source 1.tiff]

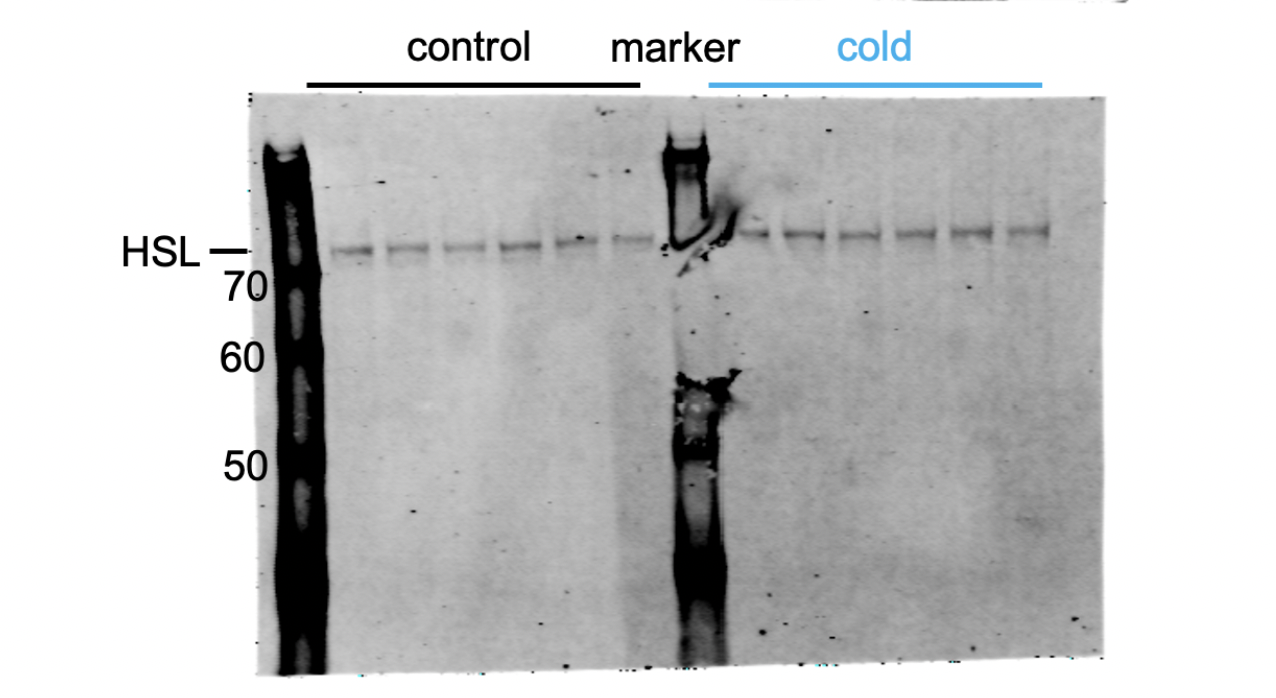

Supplement: Figure 4—source data 2. [file elife-98353-fig4-data2.zip › Figure 4A (HSL)-source 1.tiff]

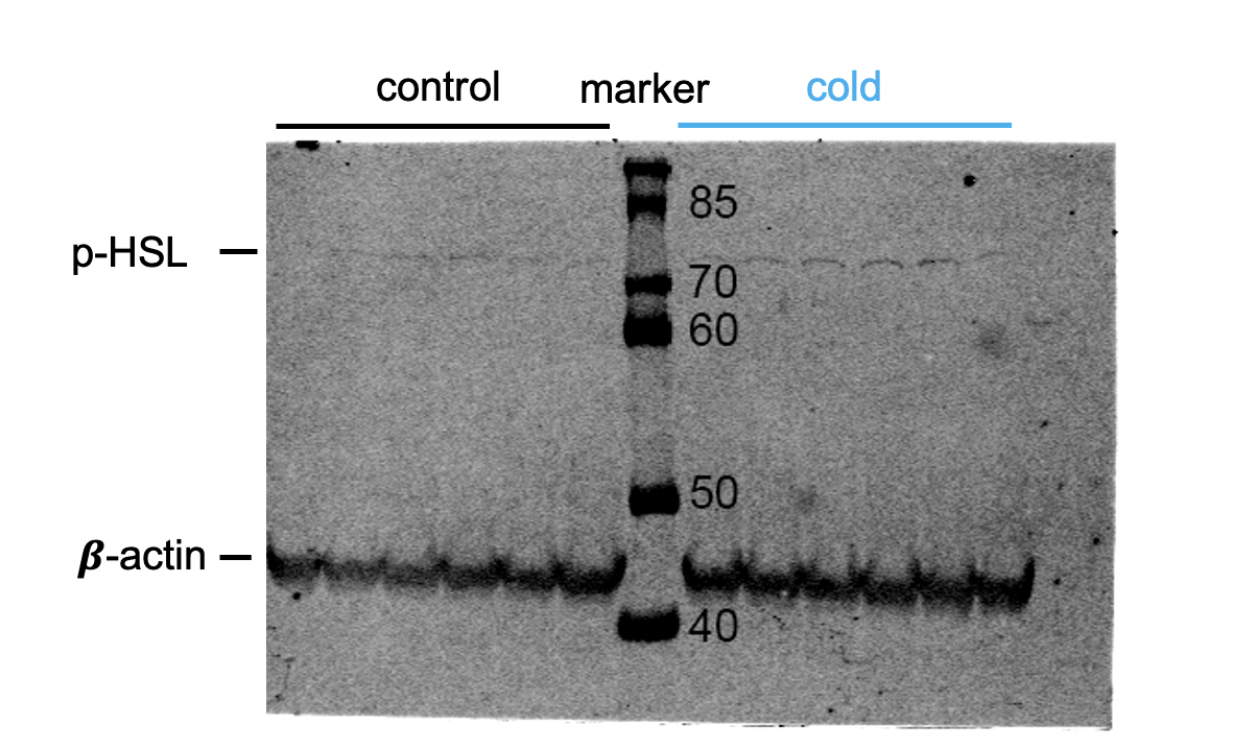

Supplement: Figure 4—source data 2. [file elife-98353-fig4-data2.zip › Figure 4A (p-HSL, actin)-source 1.tiff]
